# A Camel‐Fur‐Inspired Micro‐Extrusion Foaming Porous Elastic Fiber for All‐Weather Dual‐Mode Human Thermal Regulation

**DOI:** 10.1002/advs.202407260

**Published:** 2024-09-28

**Authors:** Yushu Wang, Zeling Wang, Hanyi Huang, Yaozao Li, Wentao Zhai

**Affiliations:** ^1^ School of Materials Science and Engineering Sun Yat‐sen University Guangzhou Guangdong 510275 China; ^2^ Nanchang Research Institute Sun Yat‐sen University No. 269 Aixihu Lake North Road Nanchang 330096 China

**Keywords:** micro‐extrusion foaming, passive radiate cooling, porous elastic fiber, thermal insulation, thermal regulation

## Abstract

Maintaining the stability of human body temperature is the basis of ensuring the normal life activities of witness, and the emergence of various functional clothing is committed to assisting the human body temperature in thermal comfort range in the changeable environment. However, achieve dual‐mode thermal regulation for cooling and insulation on an integrated material without energy input and addition of functional particles has thus far been a huge challenge. Herein, a biomimetic camel‐fur designed micro‐extruded physically foamed porous elastic fiber (MEPF) using thermoplastic polyurethane (TPU) elastomer as raw material is reported, and its dual‐layered fabric (MEPFT‐d) for effective personal thermal comfortable management at extreme temperature differences. Benefit from its micro‐nano‐pores structure, MEPFT‐d represents radiate cooling capacity by high solar reflectance and emissivity, behaves low thermal conductivity delaying heat scattering, and promotes evaporative cooling by unidirectional water transport. These excellent properties ensure that MEPFT‐d reduces heat loss in cold weather (7.2 °C higher than cotton) and blocks outside heat in hot weather (10.2 °C lower than cotton), which is suitable for various complex outdoor scenes. The cost‐effectiveness and superior wearing comfort of this work provide innovative pathways for sustainable energy, smart textiles, and personal thermal comfort applications.

## Graphical abstract

Existing personal thermal regulation materials fall short of meeting the demands for sustainable all‐climate wide range temperature management. Herein, an elastic porous fiber and its fabric are developed by micro‐extrusion physical foaming method, which not only has excellent radiation cooling performance and evaporative cooling function, but also has good thermal insulation effect. This work provides an effective strategy for mass‐prepared fabrics for personal thermal regulation of extreme temperatures.

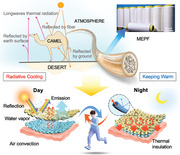

## 1 Introduction

In recent years, with global climate change and the recurrence of extreme weather, the global desert area expands accordingly, which causes the change of climate and atmospheric circulation pattern, and ultimately affects the production and life of human being.^[^ ^1^, ^2^ ^]^ According to the data published by the United Nations Convention to Combat Desertification (UNCCD), the current global area of deserts is ≈17.64 × 10^6^ km^2^, accounting for 12% of the land area.^[^ ^3^ ^]^ Even worse, the desertification land is still expanding at the rate of 50 000 to 70 000 km^2^ per year, and the proportion of global deserts is predicted to reach 20% by the end of 21st century.^[^ ^4^ ^]^ In addition to actively taking measures to reduce the rate of desertification, achieve human safety protection and improve human comfort in the complex environment has an important necessity. Although various active cooling/heating devices, such as air conditioners,^[^ ^5^ ^]^ heaters^[^ ^6^ ^]^ have been developed, they are energy intensive and do not facilitate portable. Therefore, the development of passive high‐performance thermal management textiles that can improve human thermal comfort and meet the need for energy conservation and emission reduction needs to be brought to the forefront.^[^ ^7^, ^8^, ^9^, ^10^ ^]^ Considering that heating and cooling of space consumes a large amount of energy, personal thermal management technology is a more efficient and cost‐effective alternative to directly regulate the heat exchange between the human body and the surrounding environment by endowing clothing materials with thermal management properties.^[^ ^11^, ^12^, ^13^, ^14^ ^]^

The main heat of local heating comes from the near‐infrared band of solar radiation (0.25–2.5 µm), while the radiation of heat through the atmospheric long‐wave infrared (LWIR) transmission window (8–13 um) to the cold outer space is another key factor for heat exchange.^[^ ^15^, ^16^, ^17^, ^18^ ^]^ Reasonable use of solar radiant energy, adjustment of material emissivity, reflectance, and heat transfer performance is an effective way to achieve thermal management. Recently, since daytime passive radiative cooling technology (PRC) was realized through inorganic radiation‐cooling materials. Various material designs such as multilayer photonic crystals,^[^ ^19^ ^]^ emission metamaterials,^[^ ^20^ ^]^ nanoparticle embedded structures,^[^ ^21^ ^]^ and photonic structure design films^[^ ^22^ ^]^ have been shown excellent PRC performance. However, these designs usually require complex manufacturing techniques, making them difficult to scale and costly.

Mimicking natural structures for thermoregulation is an instructive strategy for designing bioinspired materials for PRC with outstanding properties. Some creatures in nature could manipulate heat transfer through the special structure to adapt the extreme environments.^[^ ^23^, ^24^ ^]^ Zhang et al.^[^ ^25^ ^]^ prepared a porous fabric using natural silk as raw material which could achieve an average sub‐ambient temperature drop of 5.1 °C and temperature reductions of 6.0 and 8.3 °C compared with commercial silk and cotton textiles. Bai et al.^[^ ^26^ ^]^ simulated the microstructure of polar bear's hair to prepare a textile by the porous aerogel fiber with low thermal conductivity, which has good thermal insulation performance in cold environments. Tang et al.^[^ ^27^ ^]^ simulated the natural photon structure of white beetle wings, combined with the ordered pyramid array on the surface and the internal 3D layered holes to prepare a radiation‐cooled film for high efficiency PRC. Nonetheless, these current designs are for static situations, not applicable to dynamic conditions that change with the weather. It is essential to achieve a wide temperature management range, which with cooling (hot environment) and thermal insulation (cold environment) dual functions of dynamic thermal regulation materials.

Nevertheless, the reported effective cooling/heating dual‐mode materials to date all prepared with asymmetric Janus structure via coating, functional particle embedding, etc.^[^ ^28^, ^29^, ^30^ ^]^ These Janus materials require change the side of the material toward the environment to achieve radiation cooling or solar heating function to meet human body thermal comfort at different environment temperature. Zhao et al.^[^ ^31^, ^32^ ^]^ reported a multi‐layer composite fabric consisting of radiation cooling layer, thermal insulation layer, and radiation heating layer, which is used for temperature control in extreme weather. Li et al.^[^ ^33^ ^]^ prepared a passive nanostructured multilayer porous film that can be used for optional local heating and cooling in a heating/cooling mode that increases/decreases the temperature of the skin simulator by 8.1/6 °C, respectively. The results show that although the Janus structure achieves thermal regulation.^[^ ^34^, ^35^ ^]^ The optimal dynamic thermal regulation material should achieve no need to change the exposure surface adjust its thermal management performance at different ambient temperatures.

Remarkably, it is different from the radiation cooling materials represented by the current bionic silver ant, silk, beetle, etc., or the warm materials represented by polar bears and penguins.^[^ ^36^, ^37^ ^]^ Some mammals in the desert have seemingly “anomalously” evolved thick coats to maintain normal body temperatures.^[^ ^38^ ^]^ Camel as a typical example, in order to adapt the desert's high solar radiation intensity and the large temperature difference between day and night, camel has evolved a porous hollow hair fiber shape and a double coat structure.^[^ ^39^, ^40^ ^]^ The special porous fiber and double‐layered fur reflects solar radiation to cool down quickly during the day, increasing the efficiency of sweat heat dissipation, and insulates at night when the temperature drops rapidly.^[^ ^41^ ^]^ Obviously, the camel's thermoregulation system is a typical example of thermodynamic regulation. However, there is no reported relevant work on integrated thermal management materials prepared by the bionic camel thermal management system structure. According to the current research, polymers have displayed exceptional potential for radiative cooling due to their intrinsic chemical bond resonance in the infrared (IR) range for high reflection.^[^ ^42^, ^43^ ^]^ Meanwhile, rationally designing porous structures in the polymeric, limitation of pore size distribution and range is expected to obtain materials with synergistic effect of radiation regulation function and low thermal conductivity to achieve efficient personal thermal management at high and low temperatures simulating camel body temperature regulation system.^[^ ^44^, ^45^, ^46^, ^47^ ^]^

Herein, we reported a physically foamed thermoplastic polyurethane (TPU) porous elastic fiber (MEPF) and bioinspired porous fabric (MEPFT‐d), which features assembled hierarchical micro‐nano‐pores structure. The MEPF with directional and penetrating porous structures was prepared to reduce the thermal conductivity, thereby effectively enhancing the thermal insulation performance and strengthening the radiative cooling capacity of the MEPFT‐d. Inspired by the camel fur layer, we modified the hydrophilicity of the post‐stretched fibers and obtain a unidirectional water‐transporting dual‐layer cooled fabric by knitting process. In severe high‐temperature environments, the cooling performance is achieved by blocking external heat transfer, evaporative heat dissipation, reflecting sunlight, and emitting infrared radiation. Inversely, in severe low‐temperature environments, MEPFT could reduce heat loss and thermal radiation of the human body to achieve thermal insulation. In addition, excellent elastic and strong mechanical properties enable the fibers suitable for knitting and weaving, and show the excellent washing and abrasion resistance. Given these attractive advantages, this work would offer advanced insight for the development of functional textiles.

## 2 Results and Discussion

### 2.1 Working Principle of MEPF and MEPFT

**Figure 1:**
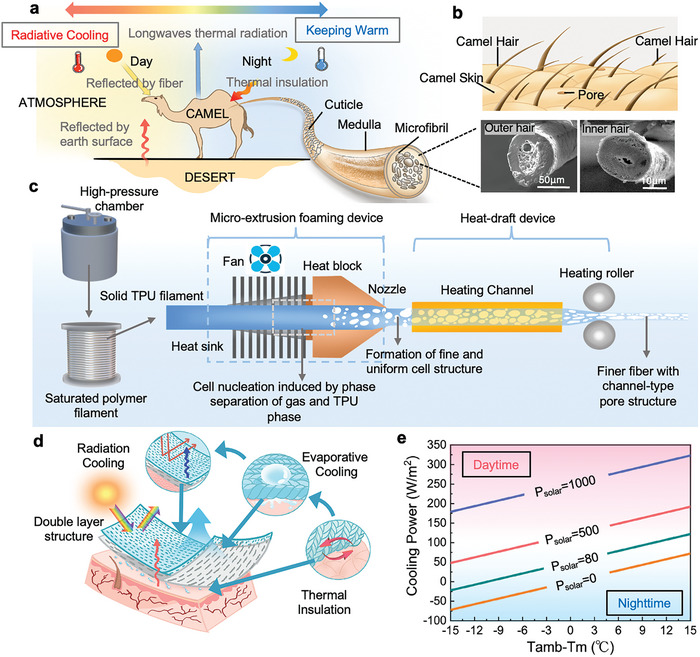
Working Principle of MEPF and MEPFT. a) Schematic diagram of the heat dissipation mechanism of camels in the desert. b) Schematic diagram of the structure of the double hair of the camel. c) Schematic diagram of preparation of MEPF by micro‐extrusion and physical foaming process. d) Schematic diagram of MEPFT thermal management mechanism. e) Theoretical calculations of the net cooling power of the MEPFT‐d in the daytime and nighttime.

In the desert region, the specific low heat capacity of sand and gravel make the surface being heated up at daytime and cooled down at night rapidly with a large temperature variation of ≈30–40 °C, while the air convection complicates the temperature variations. Camels are well known for their ability to adapt to extreme temperature changes in the desert (**Figure** **1**a). The porous hair and multiple coat layers,^[^ ^48^, ^49^ ^]^ i.e., breathable outer coat and warm inner coat (Figure 1b), are verified to be the critical elements behind the phenomenon. The porous outer hair captures a large amount of static air,^[^ ^42^ ^]^ which may provide excellent near‐infrared reflectivity and far‐infrared emissivity,^[^ ^46^ ^]^ as well as high hydrophobicity and breathability; while the porous inner hair is much thinner, and its tightly distributed structure reduces the number of heat‐conducting paths, and thus enhances thermal insulation by inhibiting heat conduction and convection. Meanwhile, camels store large amounts of water in blood vessels under the fur, which released through sweat glands throughout the skin to regulate their body temperature.^[^ ^50^ ^]^

Herein, we developed a porous elastic TPU fiber (MEPF) by a novel micro‐extrusion foaming method.^[^ ^51^ ^]^ As shown in Figure 1c, a schematic diagram of the micro‐extrusion foaming process contains the compressed CO_2_/N_2_ saturation of TPU filament, micro‐extrusion foaming of the saturated TPU filament, a post‐heat‐ stretching of the as‐prepared porous TPU fiber, and winding of porous fiber. The mixed CO_2_/N_2_ was used as a green physical blowing agent, where the CO_2_ possessed a high gas solubility, the N_2_ presented a low gas desorption rate, and an appropriate matching of CO_2_ and N_2_ pressure was suitable for achieving a stable micro‐extrusion foaming process. During the micro‐extrusion foaming process, the saturated TPU filament was continuously fed into the extruder with fixed temperature of 220 °C, the temperature‐rising induced cell nucleation and gas‐diffusion induced cell growth within the nozzle led to the formation of micro‐nano‐pore structure in the porous TPU fiber. The SEM image of the TPU fiber before the original foaming can be seen that the cross‐section of the fiber is smooth and uniform, and it is solid without pore structure, so the fiber has a macroscopic appearance of translucent, with a diameter of ≈250 µm (Figure S1, Supporting Information). As can be seen in Figure S2 (Supporting Information), the TPU fiber expands to 400 µm after foaming, and due to the introduction of the pore structure, the macro appearance of MEPF becomes opaque white, which improves its sunlight reflectivity (Figure S3, Supporting Information). With the aim to fabricate the fine porous TPU fiber, the as‐obtained porous TPU fibers were subjected to thermal stretching at 100–120 °C. Based on the stretching rates and treatment temperatures, the MEPFs with different diameters and cell structure could be prepared.

With the aim to further enhance the thermal management performance, the MEPF with different diameters was selected to weave the double‐sided fabric with an asymmetrical structure. Moreover, the MEPF with smaller diameter was modified hydrophilically, and finally the asymmetric unidirectional wetting gradient double‐layer fabric was fabricated, named as MEPFT‐d. As demonstrated in Figure 1d, the cooling mechanisms of MEPFT‐d can be divided into three aspects: 1. Radiative cooling mechanism: the coexistence of micro and nanopores enhances solar reflectivity and mid‐infrared emissivity in a wide wave‐band, thus preventing the sunlight heat during the day; 2. Evaporative cooling mechanism: the construction of the unidirectional water‐transporting fabric with layered structure can accelerates sweat evaporation, and removes the heat from the skin; 3. Thermal insulation mechanism: the porous structure endow the fabric with low thermal conductivity, which act as the role of thermal insulation and reduces the heat input. Furthermore, we calculated the theoretical net cooling power based on the solar reflection and emissivity, and investigated the radiant cooling capacity during the day and at night (Figure 1e).^[^ ^52^, ^53^, ^54^, ^55^, ^56^ ^]^ Under direct sunlight during the daytime, the net cooling power of MEPFT‐d reaches up to 300 W m^−2^, and the cooling power increases with the increase of ambient temperature. At night, because there is no solar input, and considering the cooling slowdown of the porous result, the MEPFT‐d is able to obtain supra‐ambient temperatures, 15 °C above the ambient temperature, to achieve insulation in low temperatures (the specific calculation details were provided in the Note S1, Supporting Information).

### 2.2 Microscopic Characterization of MEPF and MEPFT

**Figure 2:**
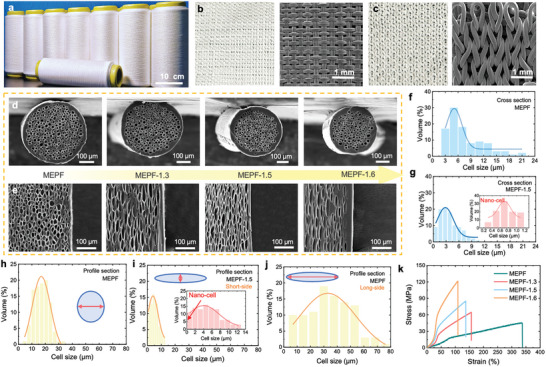
Microscopic Characterization of MEPF and MEPFT. a) Optical images of as‐prepared MEPF reels showing the potential of large‐scale fabrication. Optical image of a textile fabricated on a b) rapier loom, and c) knitted loom, indicating the potential of large‐scale production, and the SEM image showing the detail of woven structure. d) Cross‐sectional and e) Profile‐sectional microstructure SEM images of MEPF, MEPF‐1.3, MEPF‐1.5, MEPF‐1.6. f) Cell size distribution of cross‐sectional of MEPF, and g) MEPF‐1.5, illustration of the nano‐pore distribution of MEPFT‐1.5. h) Cell size distribution of profile‐sectional of MEPF, i) MEPF‐1.5 (short‐side), and j) MEPF‐1.5 (long‐side) illustration of the nano‐pore distribution of MEPFT‐1.5. k) Tensile strain curves indicating the enhanced strength with the stretching multiple.

MEPF produced by micro‐extrusion foaming method can be processed on a large scale to obtain elastic fibers of different specifications (**Figure** **2**a). Meanwhile, due to its excellent mechanical strength, MEPF can be woven on the loom and is suitable for various processes. Figure 2b shows the knitting fabric and Figure 2c shows the weaving fabric, which can be selected to meet different application needs. In order to improve the performance of the MEPF for more application scenarios, it has been thermally stretched. The microstructural morphology of MEPF with different stretching multiples is shown in Figure 2d,e. The MEPFs with stretching multiples of 1.3, 1.5, and 1.6 are named MEPF‐1.3, MEPF‐1.5, and MEPF‐1.6, respectively. According to the SEM of the fiber section (Figure 2d), the diameter of the fibers shows an obvious shrinking tendency with increasing strain multiple, from 400.0 µm (MEPF) decreases to 200.0 µm (MEPF‐1.6). The changes in the fiber profile are more specific, and the pore morphology of the fiber profile changes significantly with the increase in draw ratio (Figure 2e). Under the action of strong tensile, the pore structure gradually shows a uniform deformation orientation, and the original round pore structure is transformed into a slender pore structure, and the coexistence of micropores and nanopores is produced.

The pore distribution of the fibers before and after stretching was analyzed for further analysis of its stretching results. (Two representative fibers, MEPF and MEPF‐1.5, were selected for the main analysis). Because of the stretching action, the pore has multi‐dimensional morphological changes, so we need to analyze the cross‐section pore (Figure S4, Supporting Information) and the profile‐section pore (Figure S5, Supporting Information) separately. As can be seen in Figure 2f, the pore distribution range on the cross‐section of MEPF ranges from 3.1–21.15 µm, mainly in the range of 3.1–8.4 µm, and the whole is in the micron range, allowing heat in the near infrared band to penetrate the fiber. With the effect of stretching, the aperture of MEPF‐1.5 is significantly reduced to the nanometer range, and the distribution area is between 0 and 1.2 µm, mainly concentrated in 0.4–1.0 µm, which can block the penetration of near‐infrared heat and greatly improve its reflectivity (Figure 2g). Moreover, the main influence on the stretching of the fiber is the shape of the bubble on the profile. In Figure 2h,i, the pore distribution of MEPF and MEPF‐1.5 (short side) profiles is not much different from that of their cross sections. However, in the long side, the bubble pores gradually changed from “round” to elongated “cocoon”, and the pore distribution area of MEPF‐1.5 (long side) increased significantly, from 3.6–33.4 µm of MEPF before stretching to 6.8–76.1 µm, mainly concentrated in 9.8–31.5 µm (Figure 2j). According to the results, MEPF and MEPF‐1.5 were selected to weave the dual‐layer fabric according to the design strategy proposed to obtain MEPFT‐d, and then the aperture distribution would be multi‐peak distribution of several orders of magnitude. The wide distributions of nanopore and micron pores are ≈500 nm and ≈50 µm, respectively. In addition, because there are certain pores between the fibers of MEPFT‐d prepared by braiding process, the cell distribution diagram of MEPFT‐d is shown in Figure S6 (Supporting Information). Most of the pores are concentrated in the micron scale and do not limit MEPF due to weaving.

In addition, if used as clothing, the wearing comfort and mechanical properties also need to be considered. With the action of stretching, the fiber density also changed regularly, showing a gradually decreasing trend (Figure S7, Supporting Information). However, because the diameter of the fibers is thinner and the cell wall is obviously thinner, the tensile breaking strength of the fibers is also sacrificed correspondingly. Figure 2k shows the tensile stress‐strain test diagram of fibers with different tensile multiples, and the tensile breaking strength increases obviously with the increase of stretch multiple. A simple test as shown in Figure S8 (Supporting Information) shows that by hanging 100 g weight at both ends of 10 cm cotton fiber, MEPF fiber, MEPF‐1.5 fiber, and solid TPU fiber, the elongation of MEPF and MEPF‐1.5 fiber can reach 160% and 170% respectively, while the elongation of cotton fiber and TPU fiber is limited. Furthermore, MEPF can be easily stretched to 150% or even 200%, showing its good elasticity and elongation (Figure S9, Supporting Information). These results demonstrated that MEPF had good mechanical and elastic properties, which are conducive to weaving functional textiles such as sportswear and other applications. To characterize the tensile strain of MEPF woven into fabric, Figure S10 (Supporting Information) shows the tension‐strain curves of MEPF, MEPF‐1.5, and double‐layer MEPF‐1.5. Under the same weaving process, the tensile strength of the fabric woven by the drawn fiber is higher. The tensile breaking strength of double‐layer fabric is higher with the same drafting ratio. In addition, we repeatedly rubbed the MEPFT‐d, MEPFT‐1, and TPU fabrics 50 000 times with the Martindale wear tester, which showed no damage or fuzziness on the surface, confirming its extremely high wear resistance (Figure S11, Supporting Information).

### 2.3 Thermal Insulation Performance

MEPF is a promising candidate for lightweight thermal insulation fiber, since its porous structure can store a large amount of static air. When the external temperature is higher than the skin temperature, the MEPFT is able to block the transfer of external heat to the skin. At severely cold temperatures, it blocks the heat transfer to reduce the heat loss from the human body.

**Figure 3:**
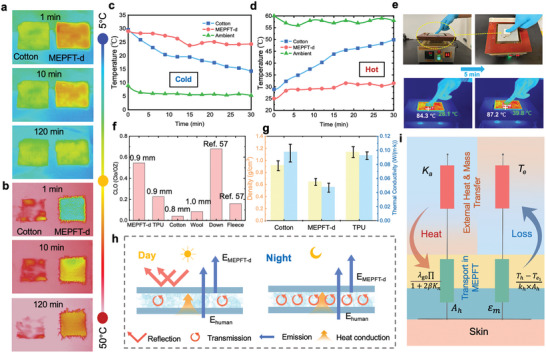
Characterization of Thermal Insulation Performance. Infrared images of a) MEPFT‐d and cotton fabrics at cold (−5 °C) and b) high temperatures (60 °C). c) Temperature variation diagram of MEPFT‐d and cotton fabric in refrigerator (−10 °C) and d) hot plate (90 °C). e) Infrared and digital photographs of the thermal insulation test of MEPFT‐d (thickness 0.8 mm) placed on a 90 °C heating plate. Within 5 min, its upper surface temperature was within the human body's tolerance range. f) Comparison of CLO values of MEPFT‐d with TPU fabrics and other common thermal textiles. g) Thermal conductivity and density of MEPFT‐d, solid TPU fabrics, and cotton fabric. h) Schematic diagram of all‐day thermal insulation mechanism of MEPFT‐d. i) Schematic diagram of MEPFT‐d thermal insulation performance model.

The thermal insulation behavior of the MEPFT‐d was investigated using an infrared camera on MEPFT‐d with the same size and thickness (≈3 mm) and cotton fabric as a control. The MEPFT‐d and cotton fabric were placed in the refrigerator, and the temperature inside the refrigerator was kept within 8 °C. The infrared images of materials are shown in **Figure** **3**a. It can be seen that the cotton fabric cooled down rapidly in the refrigerator to be similar to the ambient temperature, whereas the temperature change of the MEPFT‐d was much slower. Meanwhile, when the MEPFT‐d and cotton fabric were placed on a heating plate at 50 °C, the cotton fabric also presents a faster color change from red to white during the heating process (Figure 3b). In addition, the surface temperature covered by the MEPFT‐d always showed a significant temperature difference from the ambient temperature through the cooling and heating process, which also proved that the MEPFT‐d had excellent thermal insulation performance. Furthermore, in order to more accurately verify its thermal insulation performance as a wearable device, thermocouples were placed between the textile and the skin to test the temperature change of the microenvironment, the result showed in Figure 3c,d. After being placed in a refrigerator with an ambient temperature of 5 °C for 20 min, the temperature change of the MEPFT‐d decreased minimally to 25 °C, with a temperature difference of only 5 °C between with skin, which is still within the temperature range that is more comfortable for the human body. By contrast, the temperature difference of the cotton fabric was more obviously, with the lowest temperature dropping to 15 °C, a temperature that would clearly be felt as cold. Meanwhile, when MEPFT‐d with a thickness of 0.8 mm was placed on a 90 °C heating plate, the temperature of the non‐exposed surface in contact with the skin showed that MEPFT‐d was still within the range of human tolerance after 5 min (Figure 3e). In addition, to investigate the effect of fabric thickness on thermal insulation properties, cotton fabrics, solid TPU fabrics, and MEPFT‐d of the same height (≈2 cm) after multi‐layer stacking were placed on a heating plate at 60 °C to record temperatures, the MEPFT‐d still has a clear advantage (Figure S12, Supporting Information). To further confirm the role of MEPFT‐d in thermal insulation in cold environments, we tested its CLO value. CLO is a unit of engineering that describes the thermal insulation properties of clothing or fabric. The size of the CLO indicates how warm and insulated the fabric is. As can be seen from Figure 3f, at the similar thickness, the CLO of MEPFT‐d is 0.545, far higher than that of cotton and wool. Moreover, as a weavable fabric, its CLO value is close to that of down and fleece, which is sufficient to prove its excellent thermal performance.^[^ ^57^ ^]^ As shown in Figure 3g, the thermal conductivity of the MEPFT‐d is very low, 0.048 W m^−1^ K^−1^ measured through the experimental cross section, which is significantly lower than that of the cotton fabric (0.098 W m^−1^ K^−1^) and the solid TPU fabric (0.093 W m^−1^ K^−1^). The reduction in thermal conductivity and the good thermal insulation performance were mainly due to the successful introduction of the foam holes with their micro‐nano gradient porous structure, which was able to store a large amount of static air, thus maintaining a more balanced thermal comfort in both hot and cold conditions (Figure 3h).

To understand the thermal insulation performance of MEPFT‐d as a wearable device, we considered the heat input and output in the system, which is presented in Figure 3i. For porous materials, the thermal insulation performance is highly correlated with the heat conduction of the gas phase, in order to further evaluate the effect of the pore structure of MEPFT‐d on their thermal insulation performance.^[^ ^58^ ^]^ The heat transfer (λg) through the gas phase in MEPFT‐d is estimated as follows

(1) $$\lambda_{g}=\frac{\lambda_{g0}\prod }{1+2\beta K_{n}}$$

where *λ*_g0_ = 26.7 mW m^−1^ K^−1^ represents the gaseous thermal conductivity in free space at room temperature, *β* is a constant and its value is ≈2 for the air in ordinary pressure. *K*_n_ is the value calculated from the mean free path (*l*_m_) and pore diameter (*δ*) of air molecules. We estimated *l*_m_ in MEPFT to be 5–20 µm (the lm of air is 69 µm in the free space). The average pore sizes of MEPFT were 0.5–30.5 µm. The porosity of MEPFT is

(2) $$K_{n}=\frac{l_{m}}{\delta }$$

In addition, according to the loss of human heat can be calculated according to the following formula (The detailed parameters can be found in the Table S1, Supporting Information):

(3) $$P_{\mathrm{los}s_{1}}^{\mathrm{conductive}}=\frac{T_{h}-T_{e_{1}}}{k_{h}\times A_{h}}$$

(4) $$P_{\mathrm{los}s_{1}}^{\mathrm{radiative}}=\varepsilon_{h}\times A_{h}\times \sigma \times \left({T_{h}}^{4}-{T_{e_{1}}}^{4}\right)$$

(5) $$P_{\mathrm{los}s_{1}}=P_{\mathrm{los}s_{1}}^{\mathrm{conductive}}+P_{\mathrm{los}s_{1}}^{\mathrm{radiative}}$$

(6) $$P_{\mathrm{los}s_{2}}^{\mathrm{conductive}}=\frac{T_{h}-T_{e_{1}}}{k_{h}\times \frac{1}{2}A_{h}}+\frac{T_{h}-T_{m}}{k_{h}\times \frac{1}{2}A_{h}}-P_{\mathrm{air}}$$

(7) $$\begin{array}{c} \;P_{\mathrm{los}s_{2}}^{\mathrm{radiative}}=\varepsilon_{h}\;\times \frac{1}{2}A_{h}\times \sigma \times \left({T_{h}}^{4}-{T_{e_{2}}}^{4}\right)+\;\\ \varepsilon_{m}\times \frac{1}{2}A_{h}\times \sigma \times \left({T_{h}}^{4}-{T_{e_{2}}}^{4}\right) \end{array}$$

(8) $$P_{\mathrm{los}s_{2}}=P_{\mathrm{los}s_{2}}^{\mathrm{conductive}}+P_{\mathrm{los}s_{2}}^{\mathrm{radiative}}$$

According to the air thermal conductivity (0.023 W m^−1^ K^−1^), the human heat loss without wearing MEPFT‐d in high temperature environment (room temperature: 30 °C) is calculated to be 131.1 W. In a cold environment (room temperature: 15 °C), the human heat loss without MEPFT‐d was 293.9 W. In contrast, after wearing MEPFT‐d, the body heat loss decreased to 26.62 W per person (high temperature environment) and 133.2 W per person (cold environment). It is clear that MEPFT‐d can achieve all‐weather heat management according to the temperature difference between the external environment and the human body.

### 2.4 The Radiative Cooling Performance of MEPFT

**Figure 4:**
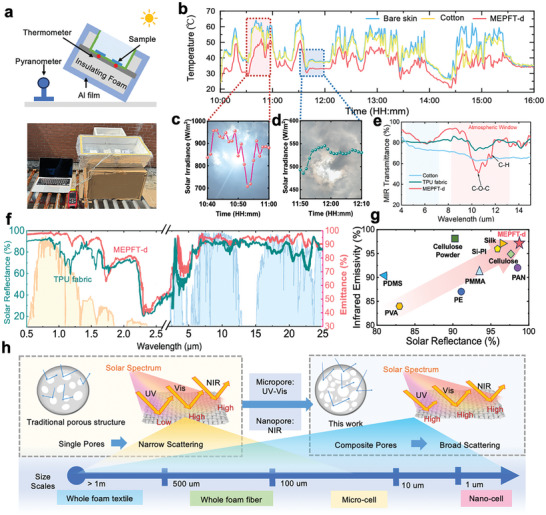
Microscopic Characterization of MEPF and MEPFT‐d. a) Outdoor radiation cooling test device diagram. b) Data charts for the results of outdoor radiative cooling tests (The samples include MEPFT‐d and cotton fabric, thicknesses were 1.1 mm and 0.98 mm, respectively). The solar radiation intensity in c) Clean sky and d) cloudy sky. (Real‐time temperature data of direct sunlight on August 4th, 2024) e) FTIR‐ATR spectra of MEPFT‐d and solid TPU fabric where the main characteristic peaks of vibrational absorption/emission in the atmospheric window. f) The spectral solar reflectance and mid‐infrared emittance of MEPFT‐d and solid TPU fabric. g) Comparison of the cooling performance of MEPFT‐d with other reported cooling materials. h) Schematic comparison of the radiative cooling principle of MEPF with that of conventional porous fibers.

In order to impede the inflow of external heat in a hot environment, high solar reflectivity and emissivity of textiles are of great significance for passive personal cooling. An experimental setup was designed to verify the outdoor cooling performance of MPEFT‐d on a hot and dry sunny day, where the arid environment was chosen to maximize the cooling potential of the atmospheric window. We chose the hottest time of the day for testing (from 10:00 to 16:00 in Guangzhou, China). The physical photos and schematic diagram of the test equipment are shown in **Figure** **4**a, and the data diagram of temperature change is shown in Figure 4b. The temperature change of the test sample fluctuates significantly with the change in solar intensity. Figure 4c shows the radiation intensity in sunny weather, reaching a maximum of 970 W m^−2^. At this time, the temperature of simulated skin covered by MEPFT‐d is lower than that of bare skin and cotton, and the maximum temperature difference can reach 10.2 °C (compared with bare skin). Although clouds block the atmospheric window in the sky (Figure 4d), making it difficult for the cooler to maintain temperatures below the environment, the simulated skin covered by MEPFT‐d is still cooler than bare skin by ≈3.7 °C. Remarkably, although the temperature of bare skin and cotton changed more with the change in solar sunshine intensity, the overall temperature of MEPFT‐d changed less. The temperature variation of the test sample fluctuates significantly with the change in solar intensity.

Based on the molecular vibration theory (molecular design at 4–25 µm in the MIR band) and the Mie scattering theory (nano and micrometer layered design at 0.3–2.5 µm in the NIR band), the existence of strong molecular vibrations is a prerequisite for the strong absorption/emission of the materials in the corresponding bands.^[^ ^59^, ^60^ ^]^ There is no obvious change in the molecular structure of the MEPFT and the TPU in macro scale, and there are only variations in the aperture size and diameter. Therefore, it can be seen in Figure 4e that the strong absorption peaks of characteristic functional groups (including C−O−C, C−H) in the atmospheric window are displayed on both sides of the MEPFT. Solid TPU and MEPFT‐d have characteristic peaks in the same position, but MEPFT‐d shows sharper peaks in the band 9.5–12.7 µm, which indicates that the introduction of pores effectively changes the path of infrared radiation from MEPFT‐d. The solar spectral reflectance (MIR 0.5–2.5 µm) and the emissivity in the atmospheric window (8–13 µm) of the MEPFT‐d bilayer fabrics and the solid TPU fabrics are shown in Figure 4f. The solar reflectance of MEPFT‐d in the near‐infrared wavelength band (0.5–2.5 µm) is 98.7%, which is slightly higher than that of the solid TPU fabrics (83.2%). Meanwhile, MEPFT‐d has excellent thermal emissivity. the thermal emissivity (97.2%) of MEPFT‐d in the far‐infrared band (16–25 µm) is significantly higher than that of the solid TPU fabric (90.3%). Notably, Figure 4g shows a comparison of the cooling performance of MEPFT‐d with other reported porous cooling materials in the literature.^[^ ^25^, ^27^, ^32^, ^33^, ^39^, ^47^, ^61^, ^62^, ^63^ ^]^ Compared to most cooling materials, MEPFT‐d demonstrates excellent solar reflectance and infrared emissivity, making it highly possible for effective passive cooling (Specific data are shown in Table S2, Supporting Information).

Compared with normal porous fiber, the mechanism of MEPFT‐d's excellent radiative cooling performance is that the pore structures in the fiber combine with the layered structure, forming a clear light scattering network and reflecting the solar spectrum in a wide‐wavelength. According to Mie scattering theory,^[^ ^64^ ^]^ when the aperture is distributed in the nanometer range, the nanopore can effectively scatter sunlight in the wavelength range of 0.3–1.5 µm. When the aperture is increased to the micron range, the scattering in other ranges is compensated (Figure 4h). Moreover, the layered porous polymer can enhance the spectral solar reflectance and improve the efficiency of radiative cooling by replacing the inorganic dielectric filler with a light‐scattering cavity. Therefore, the coexistence of micro‐nano‐pore endows MEPFT‐d a wider range of infrared radiation bands, and the selective input and output of different bands give it a stronger radiative cooling capability. In addition, Figure S13 (Supporting Information) shows the reflectance and emissivity of MEPFT‐d after repeated washing and ultraviolet irradiation, respectively. Benefiting from the excellent mechanical strength of MEPFT‐d, the fabric structure is not affected by breakage or peeling after repeated washing, so washing has no significant effect on its reflectance and emissivity. Meanwhile, TPU is a material with good UV linear resistance, and its reflectance and emissivity did not show significant attenuation after 24 h of UV irradiation.

### 2.5 Water Transportation Performance and Evaporative Cooling

**Figure 5:**
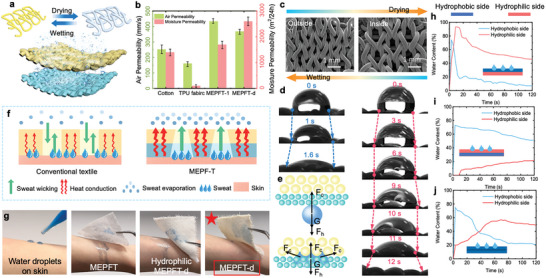
Water Transportation Performance and Evaporative Cooling. a) Double‐layer structure diagram of MEPFT‐d. b) Data chart of moisture permeability and air permeability of different fabrics. c) SEM images of hydrophilic side and hydrophobic side of MEPFT‐d. d) Contact Angle test of hydrophilic and hydrophobic sides of MEPFT‐d. e) Schematic diagram of MEPFT‐d's unidirectional water transfer mechanism. f) Schematic diagram of evaporative cooling mechanism of MEPFT‐d and traditional fabric. g) Practical test of the fast moisture‐wicking property of the MEPFT, Hydrophilic MEPFT‐d and MEPTF‐d (the blue water simulates the sweat on human skin). In a water management tester, the water content on the hydrophobic and hydrophilic sides of MEPFT‐d after water was dropped from the bottom up. h) the hydrophilic side of MEPFT‐d is below, i) the hydrophobic side of MEPFT‐d is below, j) Both the upper and lower sides of MEPFT‐d are hydrophobic.

The camel's remarkable adaptations to extreme changes in temperature are well‐known. To cope with fluctuating thermal environments, the camel stores a considerable amount of water in the blood vessels beneath its fur. The water is then released through sweat glands spread throughout the skin to regulate its body temperature. The fabric plays a crucial role in the heat exchange process between the human body and its surroundings as it serves as the primary medium for shielding the skin from the external environment. In general, the human body regulates its thermal homeostasis through various pathways for heat conduction, convection, and radiation, which work in coordination under different situations.^[^ ^65^ ^]^ While the skin temperature can easily increase surpass under conditions of excessive heat input (e.g., direct solar radiation, high humidity and high temperature environments, or states of strenuous exercise), once thermal radiation is unable to dissipate the additional heat to sufficiently effective to counteract heat input, the human core temperature will increase, subsequently leading to phenomena such as sweating. In this case, effective sweat transportation and high evaporation rates can greatly accelerate core temperature equilibrium and reduce the body's surface temperature. Thus, we partially hydrophilized the prepared MEPF and, through a knitting process, wove a layered structured fabric with unidirectional water transport properties (**Figure** **5**a). To confirm the effectiveness of this strategy, the air and moisture permeability of the prepared gradient fabric MEPFT‐d was tested, and the same tests were performed with cotton fabrics, solid TPU fabrics, and single‐layer MEPFT‐1 fabrics. According to the test results (Figure 5b), it can be seen that MEPFT‐d has excellent performance in both air permeability and moisture permeability. Due to the increase in thickness, although the air permeability of MEPFT‐d is reduced compared to that of single layer MEPFT‐1, it is still much higher than that of cotton fabric. In addition, the gradient structure design endows MEPFT‐d higher moisture permeability. During use, the hydrophobic layer is placed on the inside near the human skin, and the hydrophilic layer is placed on the outside. This strategy ensures that the skin remains dry at all times, while using capillary forces to transport sweat produced by the human skin. Thus, the outer layer is a hydrophilic treated fine fiber, and the inner layer is a hydrophobic coarse fiber (Figure 5c). As shown in Figure 5d, we detected the wettability on the both sides by measuring the static water contact angle. The droplet (volume of 1 µL) spherical shape started to collapse immediately after contacting with the fabric surface, which indicates the excellent hydrophilicity. Otherwise, the droplet maintained a spherical shape on the back side for ≈12 s and then vertically penetrated into the fabric, which shows the obvious hydrophobicity.

In Figure 5e when water droplets were in vertical contact with hydrophobic side of MEPFT‐d, the strong upward capillary force (*F*_c_) was enough to overcome gravity (*G*) and the hydrophobic force (*F*_h_) to pump water through the hydrophobic layer, which will promote water to actively penetrate into the hydrophilic layer and fully diffuse to achieve a dynamic equilibrium state. It is worth noting that the capillary force is generated by the Laplacian pressure and is negatively related to the pore radius. The wettability gradient between the two sides of the fabric determines the water transportation direction and at the same time provides an internal transport driving force, which demonstrates the different surfaces have different transport speeds for liquids, confirming the successful construction of the gradient unidirectional water transport structure. As shown in Figure 5f, When the environment is hot, the sweat on the skin can be transported from the hydrophobic inner side to the hydrophilic outer side to keep the skin dry. And when the environment becomes cold, the wettability gradient reverses, hindering the outward transport of water molecules, delaying evaporation, and reducing heat loss. Integrating Janus wettability and evaporative heat dissipation into a foamed fabric system with good thermal insulation and passive radiative cooling performance can maximize the thermal management effect of MEPFT‐d by exploiting its synergistic effect. Comparison with conventional fabrics shows that since the fabric has a gradient of wetting in addition to its fiber‐to‐fiber voids, the pore structure of the fibers also has a gradient (Figure S14, Supporting Information). Therefore, in a hot environment, the human skin will sweat along the fabric gradient, from larger pores to larger pores, generating a capillary force gradient, which sucks water from the inside to the outside, while its hydrophilic outer side further increases its transport speed to maximize the cooling efficiency. Figure 5g vividly simulates the fast transportation of simulated sweat when the fabric covers the wet skin. The blue water droplets on the skin can be instantly pumped through the fabric to the ambient environment, allowing the sweating skin to dry quickly. However, due to the lack of capillary force, single‐layer MEPFT and hydrophilic MEPFT‐d are unable to extract excessive sweat from the skin. The directional water transport capacity was further quantified by testing the water content of the hydrophobic side and hydrophilic sides of MEPFT‐d after vertically dropping simulated saline water on its top surface. When the hydrophobic side faced up (Figure 5h), the upper surface rapidly reaches its maximum water content within 10 s and then immediately drops to 20%. The water content of the bottom layer reaches 87% and then gradually decreases. Flipping the MEPFT‐d makes the hydrophilic side face up. As shown in Figure 5i, the water content at the bottom began to rise slowly after 10s, while the moisture content at the top stabilized at 76% and slowly decreased, indicating that the downward infiltration process was blocked during the test. In the Movie S1 (Supporting Information), droplets on the hydrophilic side will spread rapidly, while droplets on the hydrophobic side will gradually spread within 10 s. And after 20 s of dripping, whether the top side is hydrophilic side or the hydrophobic side, there is no water mark left on the bottom paper after removing the fabric, proving that MEPFT‐d can keep the covered area dry. In addition, we prepared a gradient pores fabric that is hydrophobic on both sides, as shown in Figure 5j. During the experiment, its decline was relatively slow and the rising trend of water content in the bottom layer was relatively gentle. Due to the double‐sided fabric without wetting gradient can only rely on the directional capillary force and gravity of the gradient pore structure to pull the brine solution from the hydrophobic layer to the hydrophilic layer.

In addition, the evaporation of water from MEPFT‐d was tested at ambient temperatures of 25 °C, 30 °C, and 35 °C (Figure S15, Supporting Information). With the increase of ambient temperature, the rate of water evaporation gradually increases. After the water is transported to the outer surface, the rate of evaporation will increase with the increase in temperature, which can provide the necessary wearing comfort in practical use and rapid drying of moist skin, reducing the risk of post‐exercise heat. The experimental results show that MEPFT‐d can realize reversible one‐way water transfer and thermal regulation with temperature change through reasonable structural design. In hot environments, fabrics can cool and dry skin by accelerating the evaporation of sweat. In the external humid environment, it can limit the invasion of water, avoid sudden cold, and ensure the thermal comfort of the human body.

### 2.6 MEPFT‐d's All‐Weather Temperature Regulation Performance

**Figure 6:**
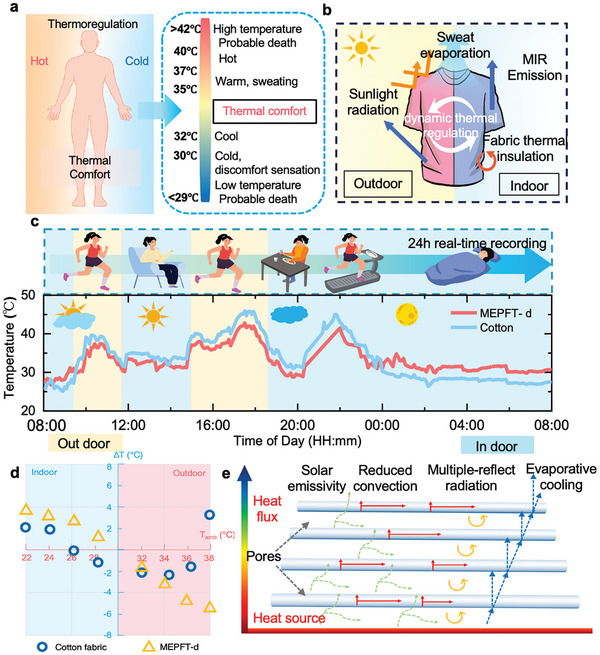
All‐Weather Temperature Regulation Performance. a) Thermal comfort range of human temperature. b) 24 h real‐time temperature recording. d) The temperature difference between MEPFT‐d and cotton and bare skin at different ambient temperatures (∆T is the bare skin temperature difference between the uncovered fabric and the covered fabric (cotton or MEPFT‐d)). e) The heat transfer mechanism of MEPFT‐d.

From the previous results, MEPFT‐d can achieve dual‐mode temperature regular in severe hot and cold environments, which can be summarized as follows: 1. Heat preservation mode: relying on the porous fiber pores and low thermal conductivity to store static air then isolate the invasion of cold air from the outside. And through the unidirectional water‐transporting fabric can stop cold and humid gases from coming into contact with the human body in rainy weather. 2. Cooling mode: The high emissivity of the TPU and the coexistence of micro‐nano‐pores give MEPF a wide infrared reflection and radiation wavelength range to achieve passive cooling. As well as through the unidirectional water‐transporting fabric can accelerate the sweat evaporation to achieve the purpose of cooling and thermal comfort. The comfortable temperature range of the human body is very limited, maintained between 30 and 37 °C (**Figure** **6**a). Therefore, it is essential to keep the temperature of the human body in a comfortable range under conditions of temperature change and constant movement of the human body. To verify the practical thermal regulation effect of MEPFT‐d, especially whether it can be used as a wearable personal temperature management device. We made a sleeve by MEPFT‐d and wore it on the arm of the experimenters for a whole day of normal activities. A temperature detector was used to record the skin temperature covered by MEPFT‐d and cotton fabric of the same thickness in real time (Figure S16, Supporting Information).

The wearable device made of MEPFT‐d described in Figure 6b has a large temperature control range in different scenarios and temperatures, which can keep the body temperature within the thermal comfort range. Therefore, Between May 23 and May 24, 2024, a long‐term temperature detection experiment was carried out in Guangzhou for 24 h. As shown in Figure 6c, the maximum temperature difference between MEPFT‐d and the ambient temperature can reach 9.2 °C and 17.9 °C between MEPFT‐d and the skin surface covered by cotton fabric during the daytime when the outdoor sun is direct, and there is no vigorous activity. When performing strenuous activities outdoors, the surface temperature of the skin after sweating reaches 34.1 °C, and the average temperature of the skin covered by cotton fabric is 36.4 °C. The average temperature of the skin covered by MEPFT‐d is 30.3 °C, and it can feel significantly cooler than cotton. In addition, in the case of no direct sunlight outside at night, the ambient temperature is relatively stable (22.0–24.0 °C). The skin temperature covered by MEPFT‐d is always ≈33.0 °C, close to the skin temperature, and less affected by wind, while the skin temperature covered by cotton fabric is very vulnerable to changes in external conditions, floating between 24.0 and 28.0 °C. In the static state of the room (indoor temperature: 23 °C), the temperature of MEPFT‐d is ≈6.0 °C higher than the ambient temperature, preventing the loss of human heat, and is in the comfortable temperature range of the human body, and the temperature of the cotton fabric is flat with room temperature. When vigorous activities are carried out indoors, the temperature difference between MEPFT‐d and cotton fabric is ≈4.0–6.0 °C, which is not as large as the temperature difference when the outdoor sun is direct, which indicates that the unidirectional water‐transporting cooling effect can work in the absence of solar radiation. Through real‐time temperature monitoring in all weather conditions, the MEPFT‐d showed high efficiency in temperature regulation in both high and cold environments, keeping the skin temperature of the covered area of the experimenters within a comfortable range (Figure 6d).

In order to further prove that its low thermal conductivity has a cold retaining function, so as to further improve its radiation cooling capacity. Temperature tests were performed on MEPFT‐d and TPU fabrics, sunproof clothing and SiO_2_‐coated foam. It can be seen that the foam with the same low thermal conductivity has a similar temperature change to MEPFT‐d, and the maximum temperature difference of sunproof clothing on the market reaches 8.2 °C (Figure S17, Supporting Information). The heat transfer mechanism of MEPFT‐d can be summarized in Figure 6e: 1. MEPFT‐d has high emissivity that could exchange heat via the atmospheric window; 2. Interference effect of micro‐nanopore improves Mie scattering and has ultra‐high solar radiation; 3. MEPFT‐d has a gradient wetting structure and unidirectional wet conductivity, which can quickly take away heat when people sweat through the woven pores and maintain a dry and comfortable skin environment; 4. Low thermal conductivity delays heat input when the external environment temperature is higher than the internal temperature, and reduces heat loss when the external environment is lower than the internal temperature. When the human body sweats, the micro‐environment between the fabric and the skin is filled with hot and humid air, while the evaporation rate of the fabric greatly affect the thermal convection, radiative cooling property affect its thermal radiation, and the low thermal conductivity affect the heat conduction of the fabric. To better understand how these modes of heat dissipation and the incident heat flow that absorbs sunlight work, we performed a theoretical analysis based on the steady‐state heat transfer model (see Note S2 and Figure S18, Supporting Information for details). This model takes into account the relationship among the fabric, the skin, and the ambient temperature where human body sweats in a hot environment. The calculation results are analyzed based on the actual test data. The results showed that when the human body exercised and sweated in an outdoor environment at 35 °C, the heat transfer of MEPFT‐d was 28.8 W, while cotton was only 14.3 W. From the results, it can be seen that the low thermal conductivity of MEPFT‐d does not affect the heat exchange in high temperature environment.

## 3 Conclusion

We report a physically foamed porous elastic fiber (MEPF), that mimics camel fur and proposes a layered cooling design fabric, MEPFT‐d, via a knitting method. The porous fiber has improved synergistic cooling with radiative thermal conditioning and evaporative cooling, as well as effective thermal insulating properties, and can be used for personal thermal management applications for effective personal cooling and thermal insulation in extremely hot and cold environments. This research integrates the ultra‐lightweight and thermal insulating properties of porous fibers with high reflectivity of 98.7% under direct sunlight and high emissivity of 97.2% due to its graded bubble size to block external heat in extremely hot environments. In addition, through the double‐sided weaving process and cross weaving of gradient wetting fibers, it forms a good unidirectional water transfer function and produces evaporative cooling effect on the body. At the radiation intensity of 970 W m^−2^, the maximum temperature difference between simulated skin covered by MEPFT‐d and bare skin can reach 10.2 °C. Even with clouds blocking the atmospheric window in the sky, the non‐exposed side of MEPFT‐d is still 3.7 °C cooler than bare skin. Meanwhile, the CLO value of MEPFT‐d is 0.545, which is significantly higher than that of common cotton, wool, and other textiles, and close to the warmth retention of down. MEPFT‐d exhibited superior air permeability, moisture permeability, and mechanical strength, and its continuous and scalable preparation method contributes to the design of sustainable and high‐performance materials for personal thermal management applications.

## 4 Experimental Section

### Materials Preparation

TPU filaments with hardness of 93 Shore A, diameter of 1.75 mm, and density of 1.22 g cm^−3^ were supplied by Fujian Guoyao New Materials, Co., China. CO_2_ with a purity of 99.9% obtained from Guangzhou Guangqi Gas Corporation was used as the physical blowing agent. Hydrophilic agent, purity 99.9%, obtained from Sinopsin Group Co., LTD.

### Preparation and Modification of MEPF

The microextrusion physical foaming process in this work uses CO_2_ and N_2_ as blowing agents. The TPU filament was saturated in 18 MPa, 30 °C mixed CO_2_/N_2_ for 24 h, and the content of the saturated mixed gas was 2%. Then the saturated TPU filament was placed in a low‐temperature environment of 5 °C with the aim to control the gas desorption rate. A home‐made micro‐extruder was used to extrude the saturated filament with the nozzle temperature at 220 °C and feeding rate of ≈4.5 m min^−1^, and the foamed porous TPU fiber was heat‐stretched at 100–120 °C with various stretching rates of 0.5‐2.5 m s^−1^. The as‐prepared porous TPU fiber was coded as MEPF. The MEPF was hydrophobically modified by using hydrophilic agent impregnation followed by drying and UV curing to obtain hydrophilic MEPF.

### Weaving of MEPFT

The automatic rapier knitting machine (Guosheng Knitting Machinery Co., Ltd.) was used for weaving, and the double‐sided fabric with different fibers on the front and back sides was knitted by double‐sided weaving method, named as MEPFT‐d. Meanwhile, the hydrophilic and hydrophobic cross‐over was chosen for the hydrophilic side of the double‐layered fabrics, to improve the unidirectional water transfer properties of the fabrics.

### Material Characterization

The morphology of porous fiber, and bionic porous structure were characterized via a COXEM EM‐30AX SEM. The specimens were quickly snapped or cut off in liquid nitrogen, glued to the sample stand with conductive glue, and then sprayed with gold on the surface. SEM was used to observe the surface, cross‐section morphology, and radial morphology of porous fibers. The moisture absorption, thermal conductivity and cooling processes of the samples were captured using thermocouples and a Nicolet 6700 Fourier Transform Infrared (FTIR) meter. The mechanical properties of the samples were characterized using a yarn strength elongometer. The contact angle of the fabrics was measured by a contact angle tester. The unidirectional water transfer capacity was measured according to GB/T21655.2‐2019 standard. The unidirectional wetting process of the fabric was filmed using a filming device. The thermal conductivity of the samples was tested using DRPL‐3B thermal conductivity tester. The density (*ρ*_f_) was calculated using the following Equation (9):

(9) $$\rho =\frac{m}{V}=\frac{4m}{\pi d^{2}h}$$

where *m* represents the mass of the fiber, *V* denotes the volume of the porous fiber, *d* refers to the diameter of the fiber, and *h* is the length of the fiber.

### Air Permeability Test

The test standard based on GB/T5453‐1997 textile fabric breathability determination. According to the breathability standard of the textile, the applied pressure was 100 Pa, the applied area was 20 cm^2^, the test temperature was 20 °C, the ambient humidity was 65%, and the air permeability of the fabric was measured ten times at different positions to take the average value.

### Weather Resistance Test

According to the standard MEPFT‐d cycle washing (GB/T 3921‐2008) and ultraviolet aging test (GB/T 3189‐2015).

### Statistical Analysis

Statistical analysis was carried out using the unpaired, two‐tailed Student's t‐test. Differences were considered significant at p < 0.05.

## Conflict of Interest

The authors declare no conflict of interest.

## Author Contributions

W.Y. and Z.W. conceived the concept of this work. W.Y. Carried out all the experiments with the assistance of W.Z., H.H., L.Y. W.Y., and Z.W. wrote the manuscript. All authors contributed to finalizing the manuscript.

## Supporting information



Supporting Information

Supplemental Movie 1

## Data Availability

The data that support the findings of this study are available from the corresponding author upon reasonable request.
