# Supplementary material for: A Camel‐Fur‐Inspired Micro‐Extrusion Foaming Porous Elastic Fiber for All‐Weather Dual‐Mode Human Thermal Regulation
Source: Adv Sci (Weinh). 2024 Sep 28;11(43):2407260. doi: 10.1002/advs.202407260 (PMC11578328; doi:10.1002/advs.202407260)
Supplement: Supplementary file 1 — Supporting Information [file ADVS-11-2407260-s001.docx]

**Supplemental Information**

**A Camel-fur-inspired** **micro-extrusion foaming porous elastic fiber for all-weather dual-mode human thermal regulation**

Yushu Wang^1^, Zeling Wang^1^, Hanyi Huang^1,2^, Yaozao Li^1^, Wentao Zhai^1,2*^

^1^ School of Materials Science and Engineering, Sun Yat-sen University, Guangzhou, Guangdong Province, 510275, China

^2^ Nanchang Research Institute, Sun Yat-sen University, No. 269 Aixihu Lake North Road, Nanchang 330096, China

*Corresponding author: Wentao Zhai (orcid.org/0000-0003-4287-4075)

E-mail: zhaiwt3@mail.sysu.edu.cn

**This PDF file includes:**

Supplementary Note S1, 2

Figures S1-18

Table S1, 2

**Supplementary Note S1. The cooling model calculation.**

A cooling model was used to simulate and estimate the cooling power of MEPFT-d, the net cooling power $P_{net}$ can be calculated by the following equation (1).

$P_{net}=P_{rad}-P_{atm}-P_{sun}-P_{non-rad}$ (1)

Where $P_{rad}$ is the emitted thermal radiation from the MEPFT-d, can be defined as equation (2).

$P_{rad}=2\pi\int_{0}^{\pi/2} \sin\theta\cos\theta d\theta\int_{0}^{\infty} \varepsilon_{m}I_{bb}(T_{m})d\lambda$ (2)

Furthermore, $P_{atm}$ indicates the absorbed thermal radiation from the atmosphere at the temperature $T_{atm}$, it can be calculated by equation (3).

$P_{atm}=2\pi\int_{0}^{\pi/2} \sin\theta\cos\theta d\theta\int_{0}^{\infty} \varepsilon_{atm}I_{bb}(T_{atm})d\lambda$ (3)

Where $I_{bb}(T)$ is the radiation intensity of black body, it can be calculated from equation (4), $c$ is the speed of light ($3\times{10}^{8}$ m🞗s^-1^), $h$is the reduced Planck constant ($1.055\times{10}^{-34}$J🞗s), $K_{B}$ is the Boltzmann constant ($1.381\times{10}^{-23}$J🞗K^-1^), and the $\lambda$ is the wavelength, respectively.

$I_{bb}(T)=\frac{4c^{2}l}{\lambda^{5}}\times\frac{1}{e^{\frac{lc}{\lambda K_{B}T}}-1}$ (4)

$P_{non-rad}$ indicates the heat transfer by convection and conduction, it can be calculated by equation (5), where $h$ is the heat transfer coefficient, $T_{atm}$ is the atmosphere temperature, and $T_{m}$ is the surface temperature of MEPFT-d.

$P_{non-rad}=h(T_{atm}-T_{m})$ (5)

**Supplementary Note S2. The heat transfer model analysis.**

A one-dimensional heat transfer model is used to determine the total heat dissipation rate of human body wearing textile of different optical properties. In this model, sunlight illumination, thermal radiation, conduction, and convection are included to simulate the heat dissipation from the body to ambient air (Figure S18).

$q=q_{rad}+q_{conv}+q_{cond}-\alpha\times q_{sun}$ (6)

For the textile covered skin, the energy balance at skin surface:

$Q=q_{rad,h}-q_{rad,m}+q_{rad,o}+q_{cond}+q_{conv}$  (7)

where *Q* is the body heat generation rate per unit area, α and *q_sun_* is sunlight absorptivity of the textile and solar irradiation power density, *q_rad, h_* is the related to the radiation heat flux from skin, *q_rad_* _,_*_m_* is the radiation heat flux from the textile, *q_rad_* _,_*_o_* is the radiation heat flux from the outer surface of textile to the ambience, *q_cond_* is the conductive heat flux in the air gap between skin and textile. All parameters are calculated with the equations (S8-S14) and Table S1

$q_{rad,h}=A_{h}\times\sigma\times T_{h}^{4}$ (8)

$q_{rad,m}=\varepsilon_{m}\times\sigma\times T_{i}^{4}$ (9)

$q_{rad,o}=2\pi\int_{0}^{\pi/2} \sin(2\theta)\int_{0}^{\infty} I_{bb}(T_{f},\lambda)d\lambda$ (10)

$q_{cond}=k_{\alpha}\times\left( \frac{T_{h}-T_{i}}{t_{a}} \right)$ (11)

Since evaporative cooling is involved, its heat convection should also take into account the convection heat transfer of hot and humid air.

$q_{conv}=h\times A\times\left( T_{o}-T_{e} \right)$ (12)

$h=Nu\frac{k_{f}}{d}$ (13)

$N_{u}=1.86\times\left( \frac{d\times R_{e}\times P_{r}}{t} \right)^{0.33}$ (14)

Where, *q_conv_* is the convection heat transfer of hot and humid air flow; *h* is the convective heat transfer coefficient of wet air flowing along the wall. *A* is the wall area in contact with the wet air; $k_{f}$ is the thermal conductivity of the fluid; d is the diameter of the pore; $R_{e}$is the Reynolds number; *P_r_* is the evaporation rate.


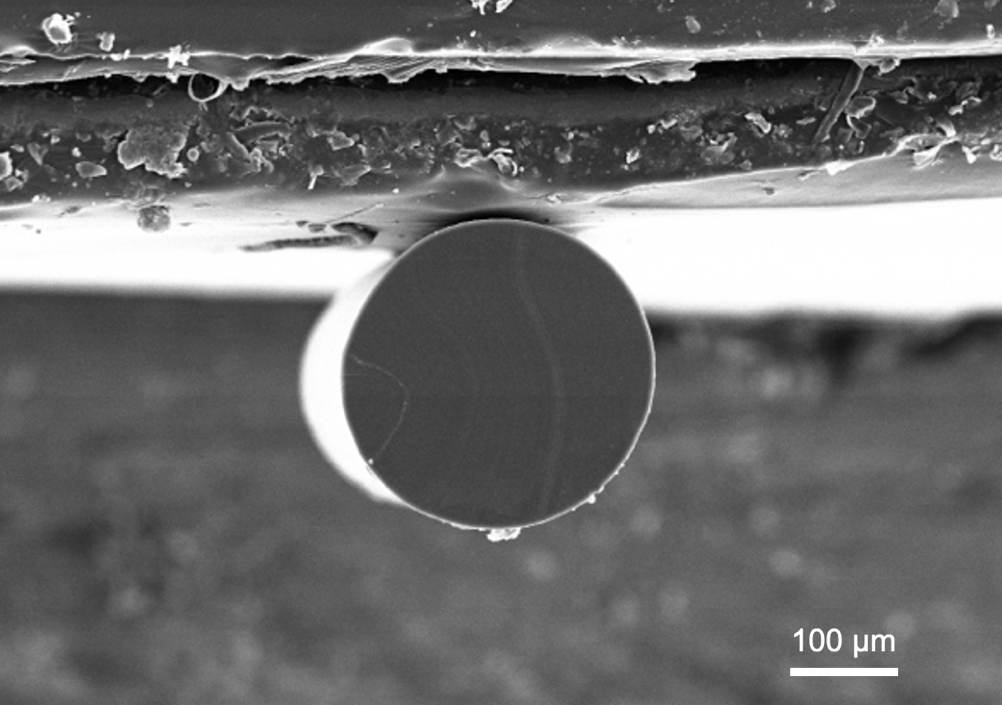


**Fig. S1** SEM image of solid TPU fiber


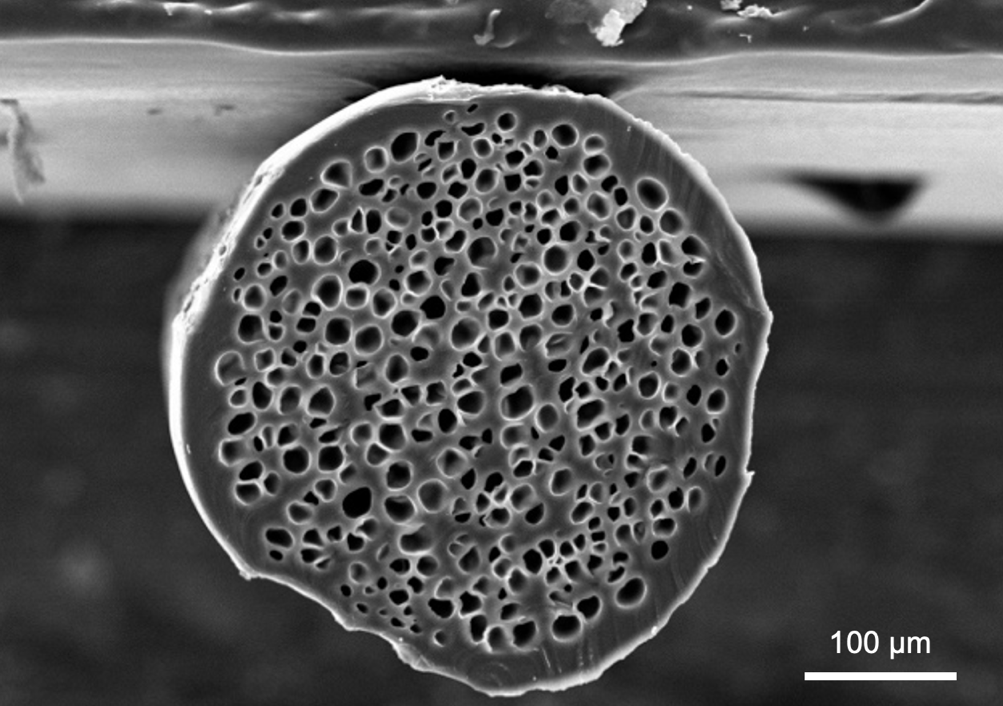


**Fig. S2** SEM image of MEPF


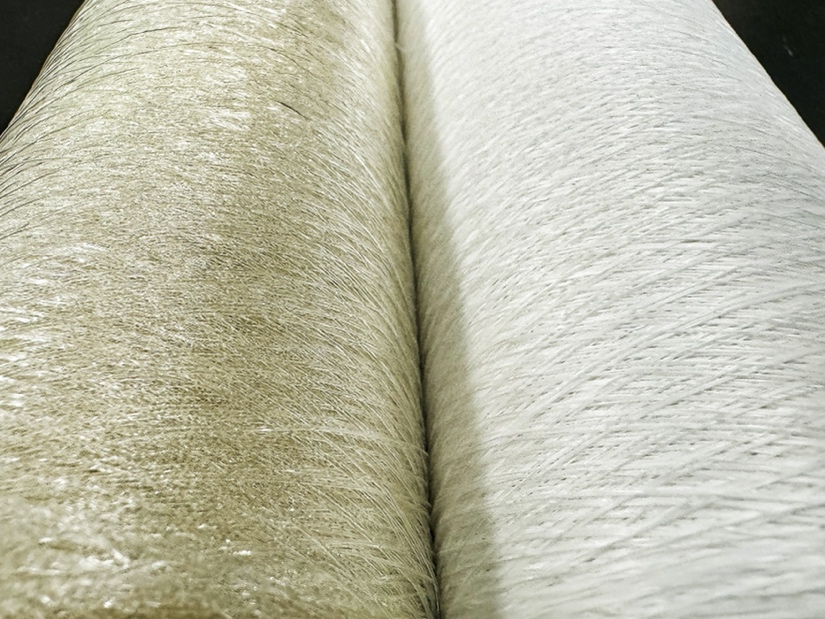


**Fig. S3** The photo of solid TPU fiber and MEPF fiber


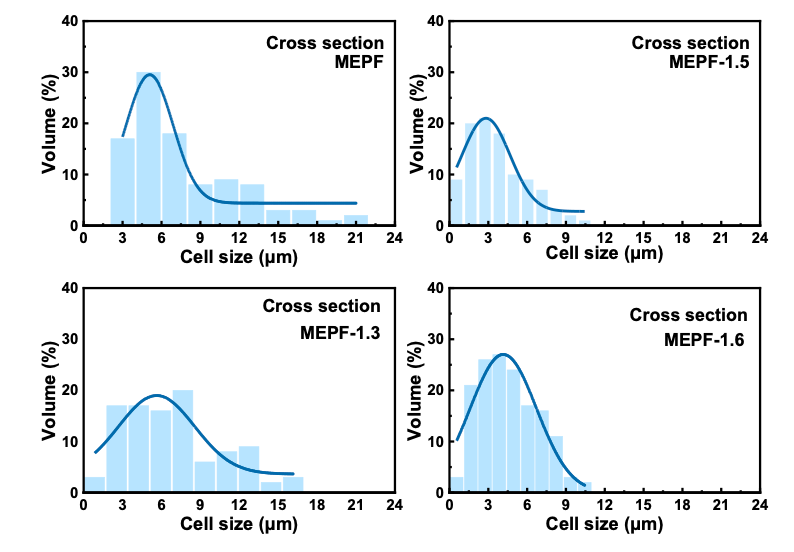


**Fig. S4** Cell size distribution of the cross-section of MEPF, MEPF-1.3, MEPF-1.5 and MEPF-1.6


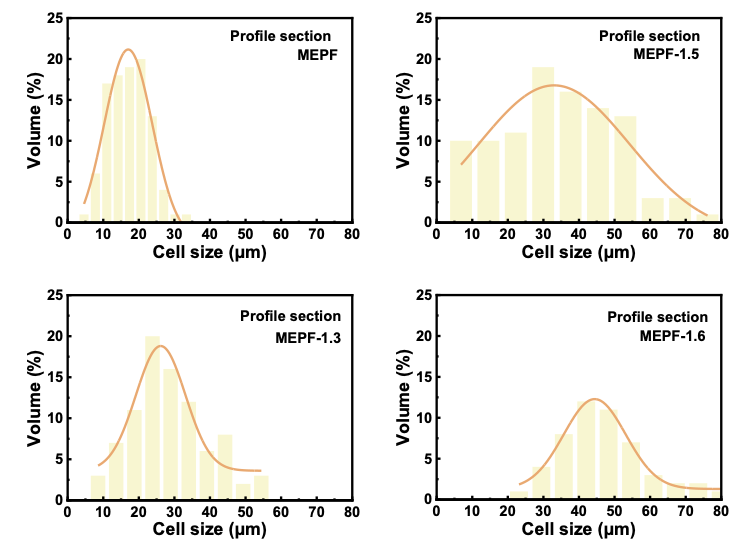


**Fig. S5** Cell size distribution of the profile-section of MEPF, MEPF-1.3, MEPF-1.5 and MEPF-1.6


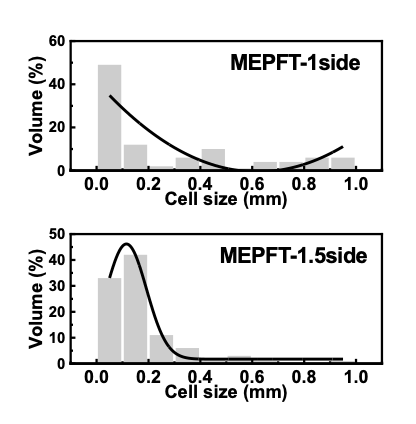


**Fig. S6** Cell size distribution of the profile-section of MEPFT-d


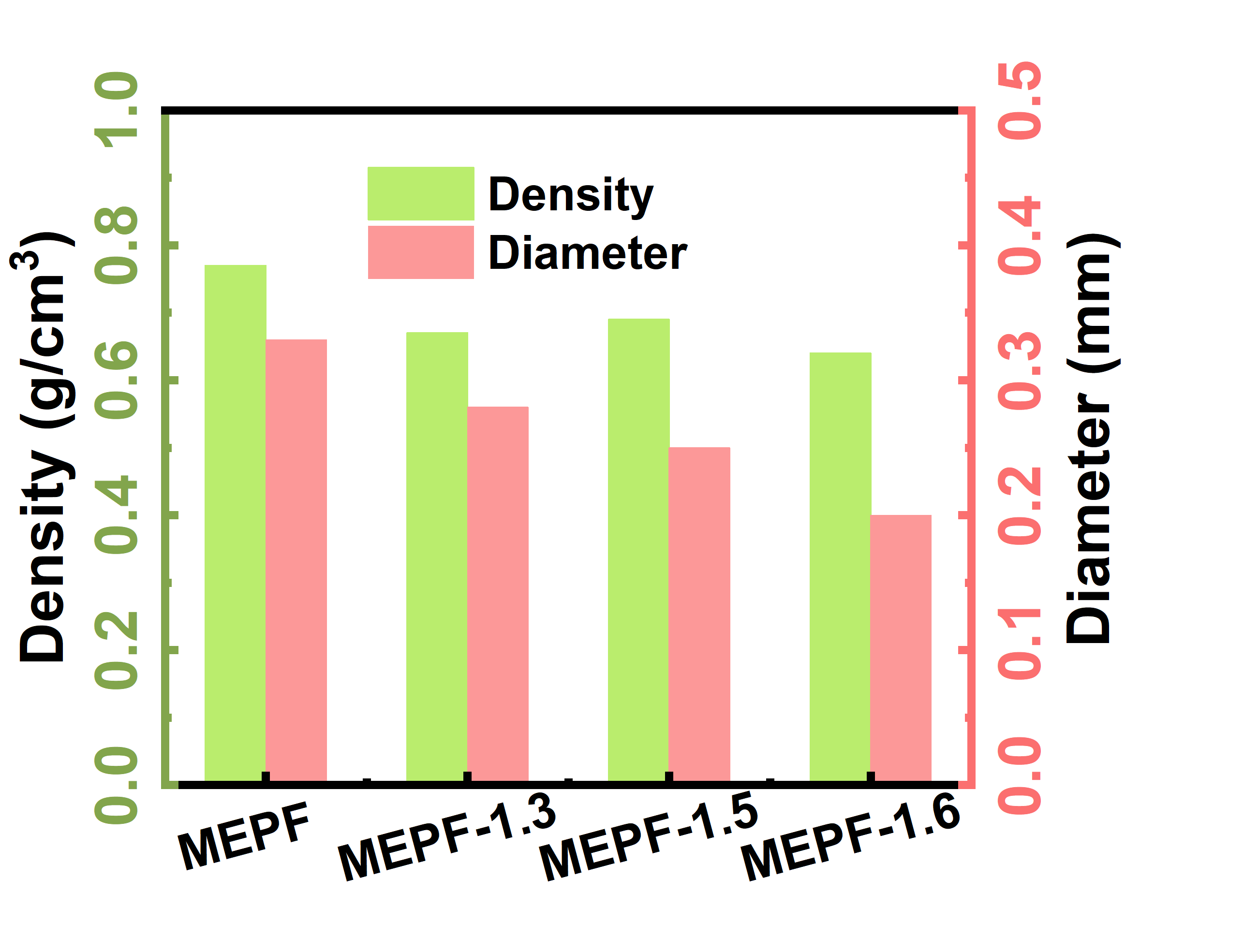


**Fig. S7** The density and diameter of MEPF, MEPF -1.3, MEPF -1.5, MEPF-1.6.


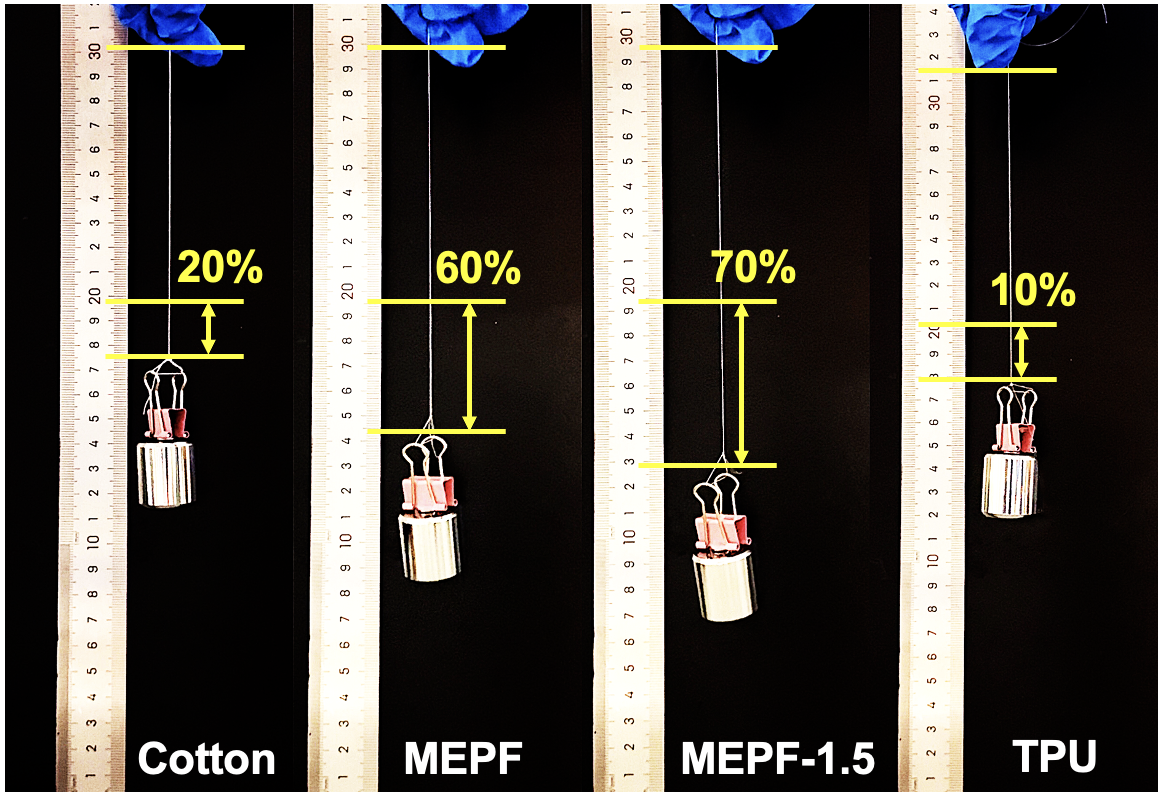


**Fig. S8** Stretching behavior of cotton fiber, MEPF, MEPF-1.5, solid TPU fiber under a weight loading of 100g


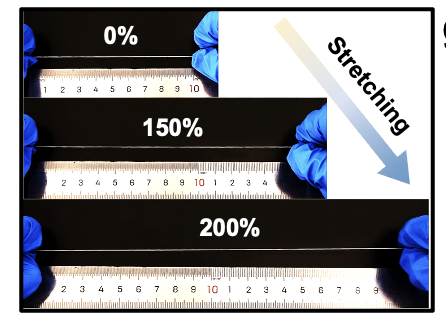


**Fig. S9** Tensile test diagram of MEPF


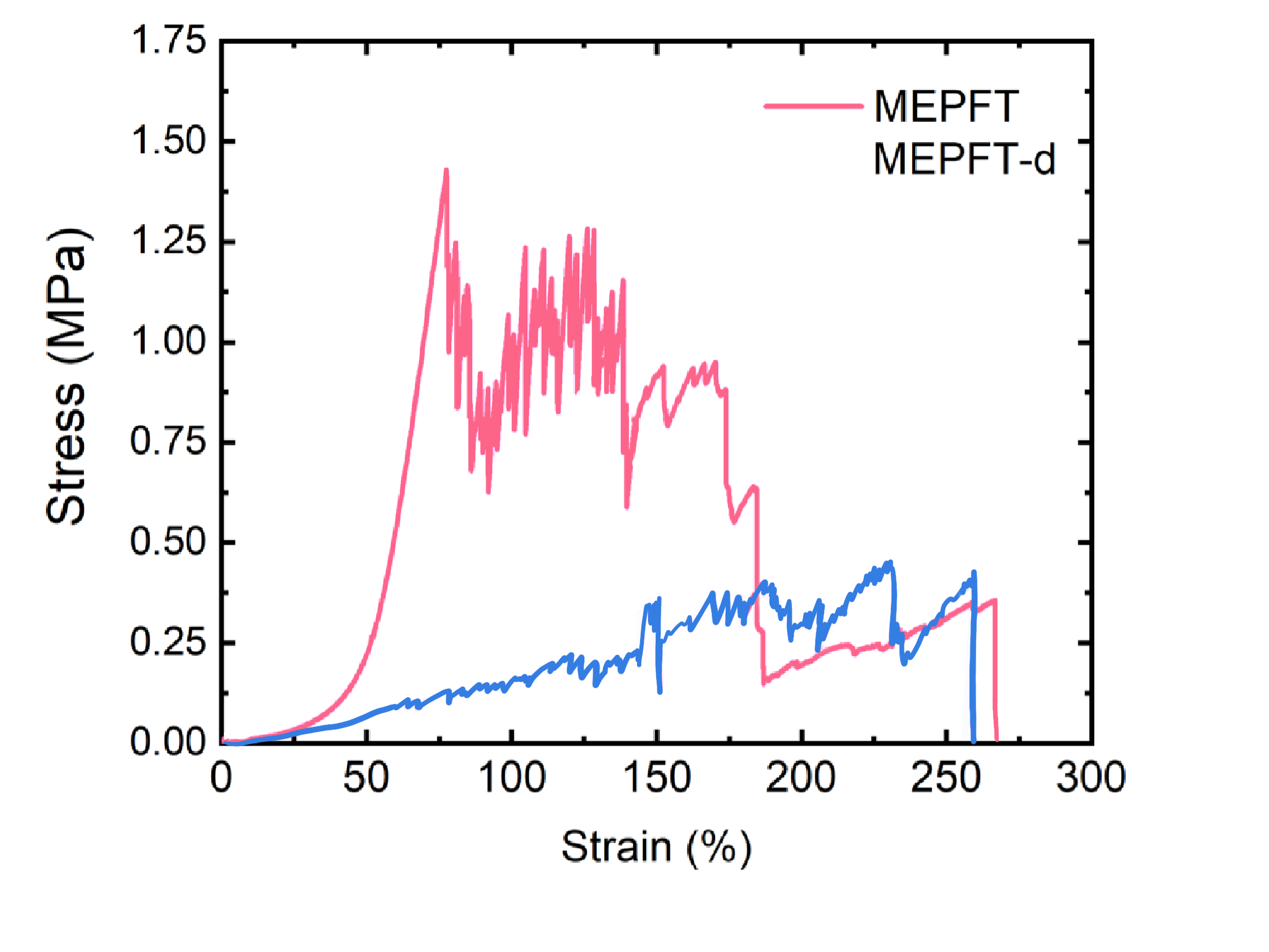


**Fig. S10** Tensile breaking strength of MEPFT-d and single-layer MEPFT.


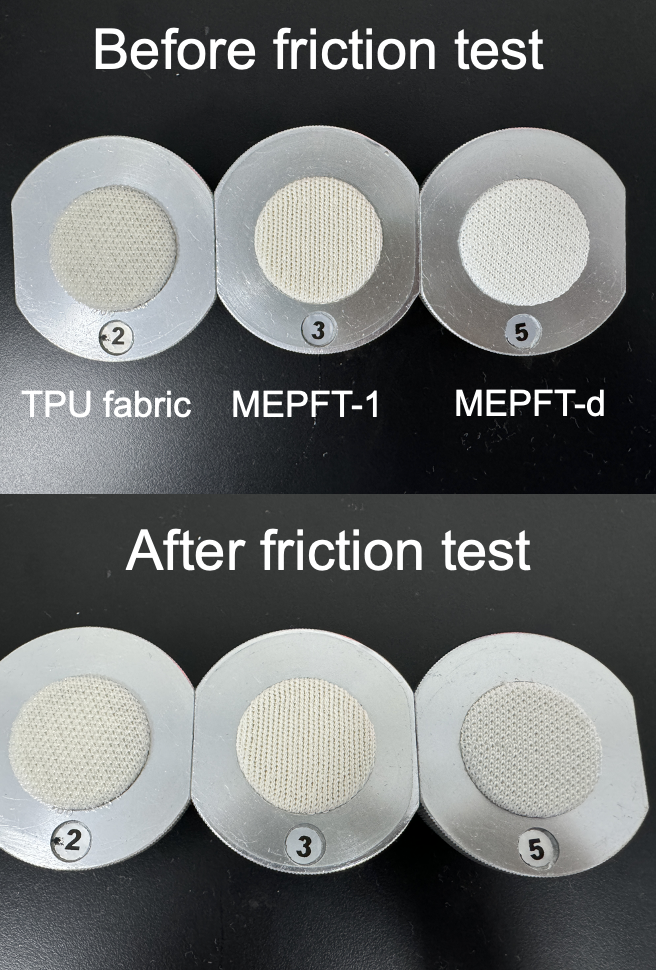


**Fig. S11** Martindale abrasion resistance test for TPU fabrics, single layer MEPFT and MEPFT-d


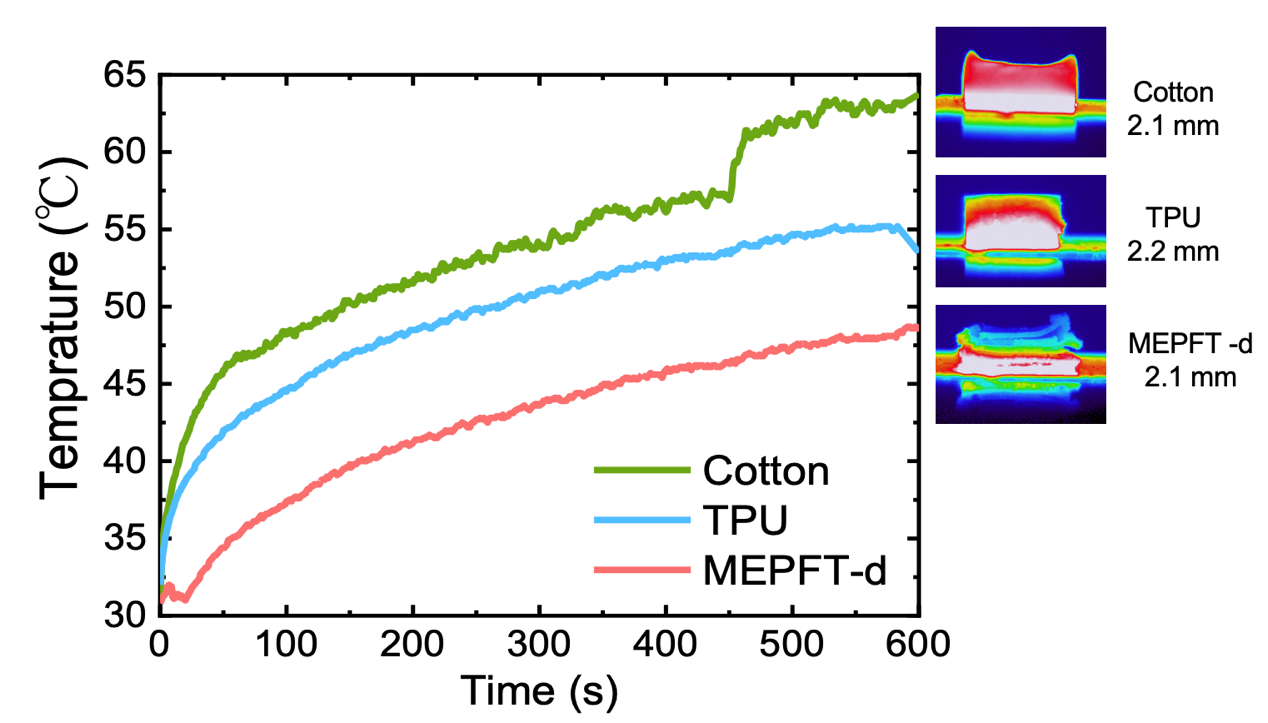


**Fig. S12** Temperature variation diagram of cotton fabrics, solid TPU fabrics and MEPFT-d (height are all approximately 2 cm) on the heating plate.


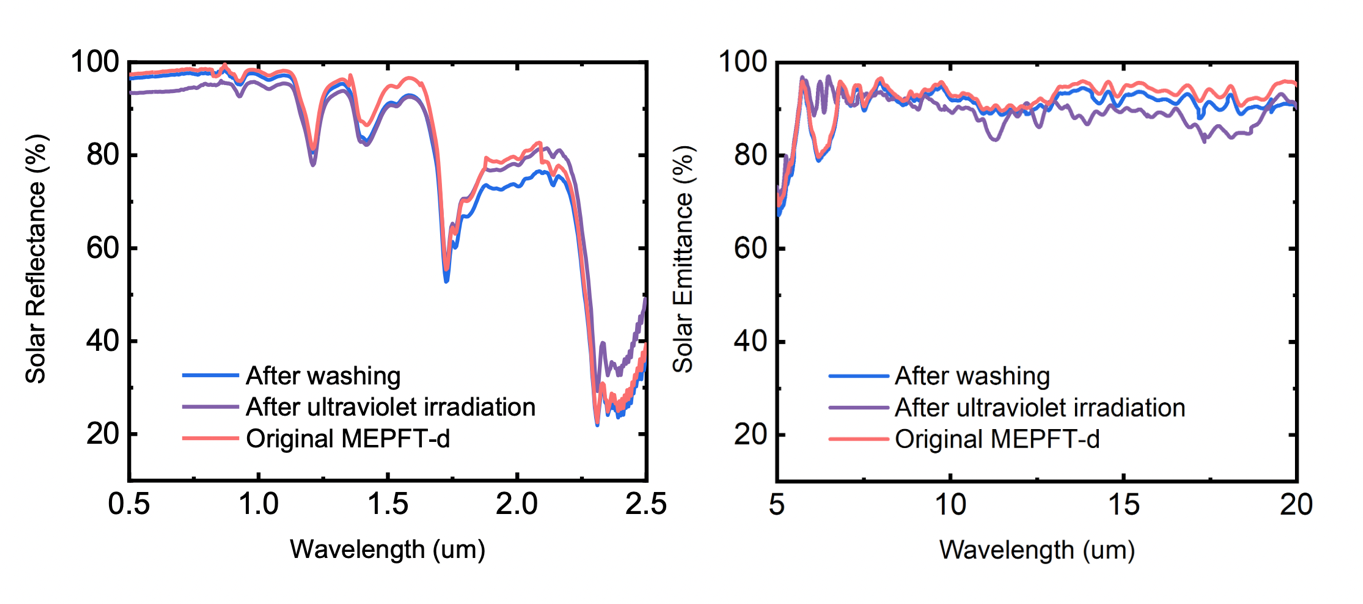


**Fig. S13** Solar reflectance and emissivity of MEPFT-d after washing and ultraviolet irradiation


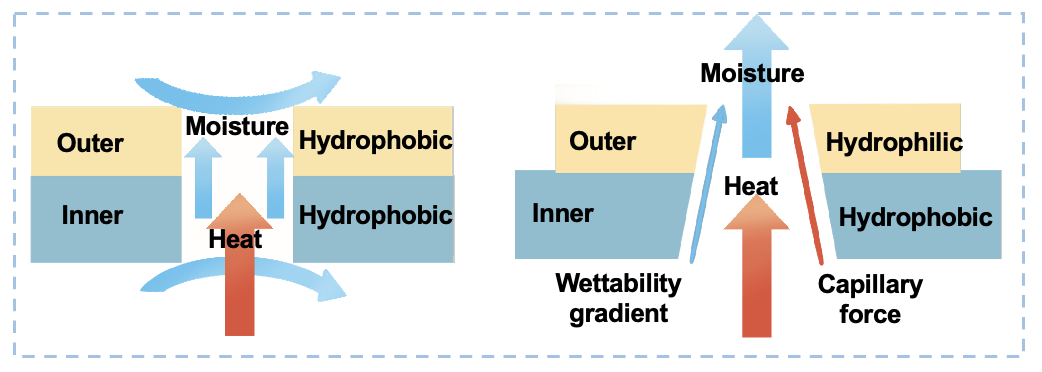


**Fig. S14** Schematic diagram of evaporative cooling mechanism of traditional fabric and MEPFT-d.


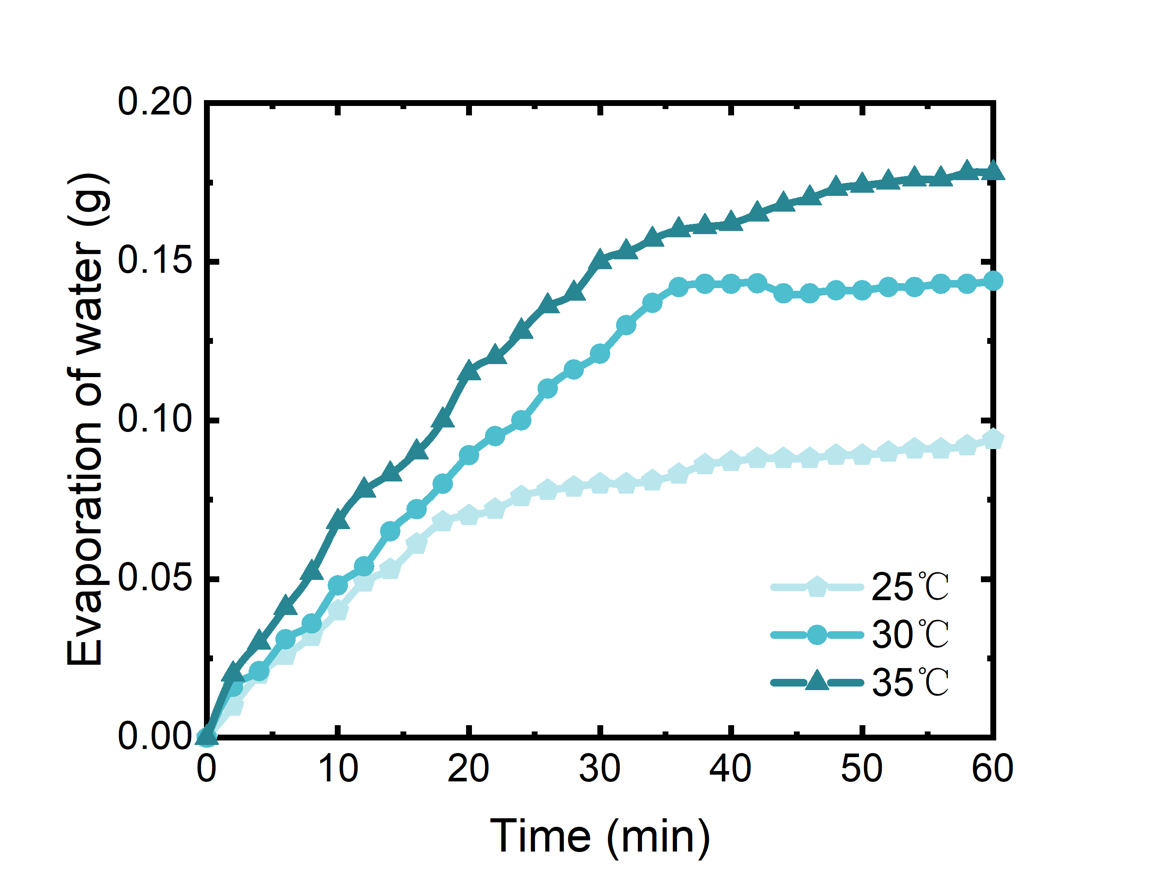


**Fig. S15** The evaporation rate of the sample was measured at room temperature 25°C, 30°C and 35°C.


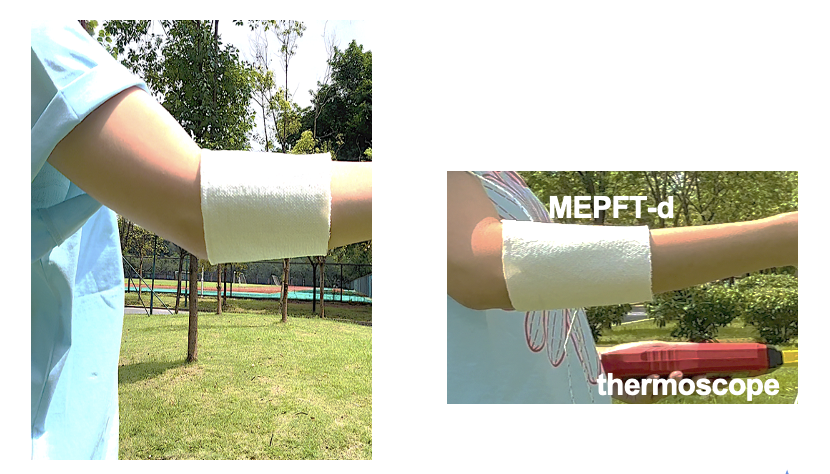


**Fig. S16** The diagram of real-time temperature detection equipment and environment.

**Fig. S17** Temperature tests were performed on MEPFT-d and TPU fabrics, sunproof clothing and SiO_2_-coated foam.


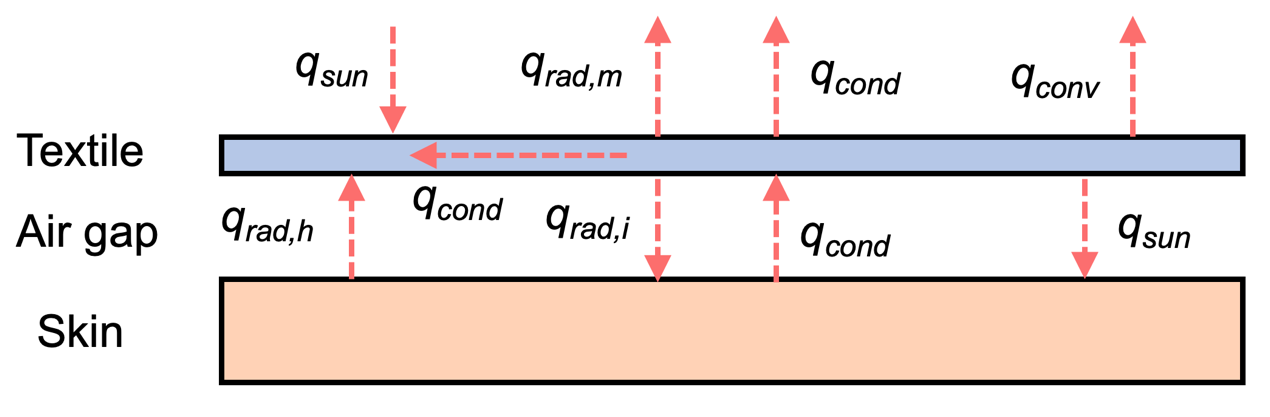


**Fig. S18** Schematic for one-dimensional steady state heat transfer model.

**Table S1** Parameters used in the analysis for the heat transfer model

| Symbol | Definition | Value | Unit |
| --- | --- | --- | --- |
| k | Thermal conductivity | MEPFT-d, k_m_= 0.048  Air gap, K_a_= 0.023  Human, K_h_= 0.432  fluid, K_f_=0.028 | [Wm^-1^K^-1^] |
| A | Area | MEPFT-d, $A_{m}=1$  Human, $A_{h}=2$ | $[m^{2}]$ |
| $\varepsilon$ | IR emissivity | MEPFT-d, $\varepsilon_{m}=0.977$  Human, $\varepsilon_{h}=1$ | unitless |
| $r_{MIR}$ | IR reflectance | Cotton, 0.63  MEPFT-d, 0987 | unitless |
| $\sigma$ | Stefan-Boltzmann constant | 5.67$\times{10}^{-8}$ | [$w/m^{2}K^{4}$] |
| T | Temperature | Environment, $T_{e1}=30$  $T_{e2}=10$  Fabric inner surface, T_i_  Fabric outer surface, T_o_  Human, $T_{h}=33$  MEPFT-d, $T_{m}=45$ | [℃] |
| t | Thickness | MEPFT-d, t_m_= 10  Air gap, t_a_= 5 | [mm] |
| R_e_ | Reynolds number | 1058 | unitless |
| P_r_ | Evaporation rate | MEPFT-d, 0.03  Cotton, 0.012 | [m^2^/s] |
| d | Pore diameter | MEPFT-d, 0.6 | [mm] |

**Table S2** Comparison of the cooling performance of MEPFT-d with other reported cooling materials in the literature.

| **Sample** | **Solar Reflectance**  **(%)** | **Infrared Emissivity**  **(%)** | | **Reference** |
| --- | --- | --- | --- | --- |
| MEPFT-d | 98.7 | 97.2 | This work | |
| PAN | 98.4 | 92.0 | 63 | |
| PVA | 83 | 84 | 39 | |
| Si | 98 | 96 | 27 | |
| PDMS | 80.1 | 90.4 | 32 | |
| Silk | 96.5 | 97.1 | 25 | |
| PE | 0.91 | 0.87 | 33 | |
| cellulose | 97.6 | 0.95 | 47 | |
| cellulose powder | 90.2 | 98.1 | 61 | |
| PMMA | 93.5 | 91.2 | 62 | |
